# Aggregation process of drifting fish aggregating devices (DFADs) in the Western Indian Ocean: Who arrives first, tuna or non-tuna species?

**DOI:** 10.1371/journal.pone.0210435

**Published:** 2019-01-15

**Authors:** Blanca Orue, Jon Lopez, Gala Moreno, Josu Santiago, Maria Soto, Hilario Murua

**Affiliations:** 1 AZTI-Tecnalia, Pasaia, Gipuzkoa, Spain; 2 Inter-American Tropical Tuna Commission (IATTC), La Jolla, California, United States of America; 3 International Seafood Sustainability Foundation (ISSF), Washington DC, United States of America; 4 Instituto Español de Oceanografía, Madrid, Spain; Hawaii Pacific University, UNITED STATES

## Abstract

Floating objects drifting in the surface of tropical waters, also known as drifting fish aggregating devices (DFADs), attract hundreds of marine species, including tuna and non-tuna species. Industrial tropical purse seiners have been increasingly deploying artificial man-made DFADs equipped with satellite linked echo-sounder buoys, which provide fishers with information on the accurate geo-location of the object and rough estimates of the biomass aggregated underneath, to facilitate the catch of tuna. Although several hypotheses are under consideration to explain the aggregation and retention processes of pelagic species around DFADs, the reasons driving this associative behavior are uncertain. This study uses information from 962 echo-sounder buoys attached to virgin (i.e. newly deployed) DFADs deployed in the Western Indian Ocean between 2012 and 2015 by the Spanish fleet (42,322 days observations) to determine the first detection day of tuna and non-tuna species at DFAD and to model the aggregation processes of both species group using Generalize Additive Mixed Models. Moreover, different seasons, areas and depths of the DFAD underwater structure were considered in the analysis to account for potential spatio-temporal and structure differences. Results show that tuna species arrive at DFADs before non-tuna species (13.5±8.4 and 21.7±15.1 days, respectively), and provide evidence of the significant relationship between DFAD depth and detection time for tuna, suggesting faster tuna colonization in deeper objects. For non-tuna species, this relationship appeared to be not significant. The study also reveals both seasonal and spatial differences in the aggregation patterns for different species groups, suggesting that tuna and non-tuna species may have different aggregative behaviors depending on the spatio-temporal dynamic of DFADs. This work will contribute to the understanding of the fine and mesoscale ecology and behavior of target and non-target species around DFADs and will assist managers on the sustainability of exploited resources, helping to design spatio-temporal conservation management measures for tuna and non-tuna species.

## Introduction

Floating objects in the surface of the tropical and subtropical oceans, also known as drifting fish aggregating devices (DFADs), tend to aggregate pelagic species underneath, including main commercial tropical tuna species (i.e. skipjack *Katsuwonus pelamis*, yellowfin *Thunnus albacares*, and bigeye *Thunnus obesus*) but also non-target species (e.g. rainbow runner *Elagatis bipinnulata*, silky shark *Carcharhinus falciformis*, dolphinfish *Coryphaena hippurus*, sailfish *Istiophorus platypterus*, green turtle *Chelonia mydas*) [1]. Generally, DFADs are composed by a floating structure (e.g. bamboo rafts with purse seiner corks) and an underwater part suspended below the floating object (e.g. nets, often tied into “sausages”, ropes, palm leaves, weights). The length of the underwater structure, which is ocean and fleet-specific, is used to reduce the drifting speed of the FAD [2] and is thought to act as a shelter for some of the associated non-tuna species [3–5]. The reasons driving this associative behavior are not fully understood, although several hypotheses are under consideration to explain the aggregation and retention processes of pelagic species around floating objects. The two most accepted hypothesis for tuna aggregation behavior are the “indicator-log” [6] and the “meeting point” hypothesis [7]. The first is based on the assumption that tuna may use floating objects as a result of an evolutionary process, since natural objects could historically be accumulated in rich waters and frontal zones and thus, be indicators of productive areas. The latter relies on the social behavior of tuna and suggests that floating objects could act as meeting points, to form and re-structure schools of tuna in an otherwise visually-void environment.

Taking advantage of this associative behavior, the industrial tropical tuna fisheries have been increasingly using DFADs in the Indian Ocean since the mid-1980s to improve catches of target species [8, 9]. The industrial purse seine catches represent around 64% of global tropical tuna catches [10]. Currently, around 50% of purse seiner total tuna catches are caught on DFADs, exceeding 70% in some years in the Indian Ocean [11, 12]. DFAD fishing represent certain notorious advantages when comparing with fishing on unassociated schools (also called free-swimming schools, FSC). For example, in the Indian Ocean the proportion of success on FAD-sets was 94% while the proportion of successful FSC sets was 58% for the Spanish fleet over the period 1990–2015 [13] and the time devoted to search for tuna schools is also reduced [8, 14]. However, DFADs may also have potential negative effects [15–17], such as higher bycatch ratios of certain species or the increase of small bigeye and yellowfin tuna catch [18], which may affect the yield per recruit. Moreover, the use of DFADs could alter the natural movements of the species associated with the objects (i.e. Ecological Trap [16]) [11, 19].

Although several works have attempted to characterize the ecology and behavior of species at DFADs [20–24], there is little information about the factors driving the mechanisms behind the aggregation process. Understanding how tuna and non-tuna species aggregate at DFADs could help to make the fishery more selective and provide information to derive bycatch mitigation, conservation and management measures. For example, it is known that fishing on larger schools can reduce the bycatch ratio [25].

Today, DFADs are equipped with satellite linked echo-sounder buoys [14], which remotely provide fishers in near real time with accurate geolocation of the object and a rough estimate of the biomass underneath. In recent years the potential use of DFADs as scientific platforms has been highlighted by the scientific community [26, 27]. This acoustic information could allow scientists to better understand the aggregation process of tuna and non-tuna species at DFADs.

This study aims to investigate the aggregation process of virgin (i.e. newly deployed) DFADs in the Western Indian Ocean using the biomass acoustic records provided by fishers’ echo-sounder buoys. For this purpose, we determine the first detection day of tuna and non-tuna species at DFAD and identify the potential differences in the aggregation patterns in relation to the depth of the underwater structure of the DFAD. Also, to better understand the potential factors involved in the aggregation process of different species groups, we examine the spatio-temporal dynamics of their biomass. All this information could contribute to develop specific management measures with the objective of reducing bycatch and, hence, assist tuna Regional Fisheries Management Organizations (t-RFMO) in the sustainable management of the DFAD fishery.

## Materials and methods

### Data collection

The acoustic data were collected through Satlink buoys (SATLINK, Madrid, Spain, www.satlink.es), which were deployed at sea by a Spanish purse seine fishing company (Echebastar fleet). The buoy contains a Simrad ES12 scientific echo-sounder that transmits the estimated amount of biomass (in tons, t) underneath the object by an Inmarsat satellite connection. Beam angle is 40⁰. The depth observation range extends from 3 to 115 m, with a blanking zone between 0 and 3 m to avoid the potential near field effect of sampling, and it is composed of ten homogeneous layers with a resolution of 11.2 m. The echo-sounder operates at a frequency of 190.5 kHz with a power of 140 W and is programmed to operate for 40 seconds. During this period, 32 pings are sent from the transducer and an average of the backscattered acoustic response is computed and stored in the memory of the buoy. Satlink buoys convert raw acoustic backscatter [28] into biomass in tons, using exclusively an empiric algorithm based on the target strength and weight of skipjack tuna, which is the main target species of the DFADs purse seine fishery. Based on experimental evidence from tagging and acoustic surveys around DFADs in the Indian Ocean [29–34], we established a virtual vertical depth limit in 25 meters as the potential boundary between non-tuna species and tuna species. Although overlap may exist between these two groups, a clear separation between tuna and non-tuna species under the DFADs have been identified at around in previous studies conducted in the Indian Ocean using a variety of information sources (i.e. tagging, scientific acoustic surveys, visual surveys) [33–35]. Thus, signals corresponding to depths shallower than 25 m (i.e. the sum of the first two layers) were assumed to correspond to non-tuna species and those deeper than 25 m (i.e. the sum of the third to the tenth layer) were assumed to be tuna. Moreover, other scientific studies using the same echo-sounder buoy in the Indian Ocean have also used similar depth limits to separate tuna and non-tuna species [24, 36]. Also, the buoy has an internal detection threshold of 1 ton of estimated tuna biomass.

Aside the acoustic information, buoy dataset includes the identification code, spatial information (i.e. latitude and longitude), and the date and GMT hour of the sampling. All this information was available for 7,514 buoys (994,065 biomass acoustic samples), which were active in the Indian Ocean from January 2012 to May 2015. Data cleaning was carried out following the next steps: i) remove data with invalid positions (e.g. positions on land or in other oceans); ii) remove duplicate records; iii) remove data with speed values higher than 3 knots (kn; likely representing onboard positions); iv) remove possible false positives produced by the detection of the DFAD underwater structure (i.e. several layers give the maximum value that a layer can have; 63t); v) remove data in the continental shelf (i.e. shallower than 200 m) as acoustic samples in < 200 m waters could provide false positives. The number of buoys and acoustic records available after the cleaning process was 5,167 and 522,964, respectively. If more than one acoustic record was available for the same buoy and day, we selected the maximum biomass acoustic signal from the buoy to avoid possible measuring variability and sampling constraints as well as obtain the best representation of daily community size.

### Identification of virgin DFADs

FAD and fishing logbooks were collected for the vessels that deployed the DFADs and the period considered in the present study. FAD logbooks provided information on the activity associated to the DFAD (i.e. deployment, fishing, visit, etc.), the object characteristics (i.e. structure dimensions, depth of the underneath part, materials, etc.), as well as buoy identification code, location and time of the activity on the DFADs. DFADs can be newly deployed (i.e. virgin FADs), transplanted (i.e. change of buoy) or re-deployed (i.e. returned to the water after a set or retrieved and deployed in another location). Only newly deployed DFADs were considered in this study, which were identified in the FAD logbooks and linked to our initial buoy database based on buoy identification code and date. This first match identified 1622 new deployments. Buoys that were deployed on natural objects were excluded from the study as these objects were previously in the water and their time at sea could not be accurately determined. As such, a total of 962 deployments of virgin DFADs with their posterior trajectories and biomass information were finally identified and considered.

Buoy data and FAD and fishing logbook information were crossed to assure that no fishing activity occurred in our virgin DFAD. Potential fishing sets were identified on the logbooks based on the information of buoy code, position and date. If a particular fishing set was identified to occur during the DFAD trajectory, the buoy information after the set was eliminated. Accurately, 15 sets were made on the available deployments. Also, and because fishers deactivate and change the buoy when encounter a not-owned DFAD of interest (regular fishing strategy of the purse seine fleets operating in the Indian Ocean), we can assume that the considered DFADs were not fished by vessels of other companies during our data acquisition period. If that would happen, the original buoy associated to the virgin DFAD will stop emitting information and, then, it will not be included in our analysis.

**Fig 1:**
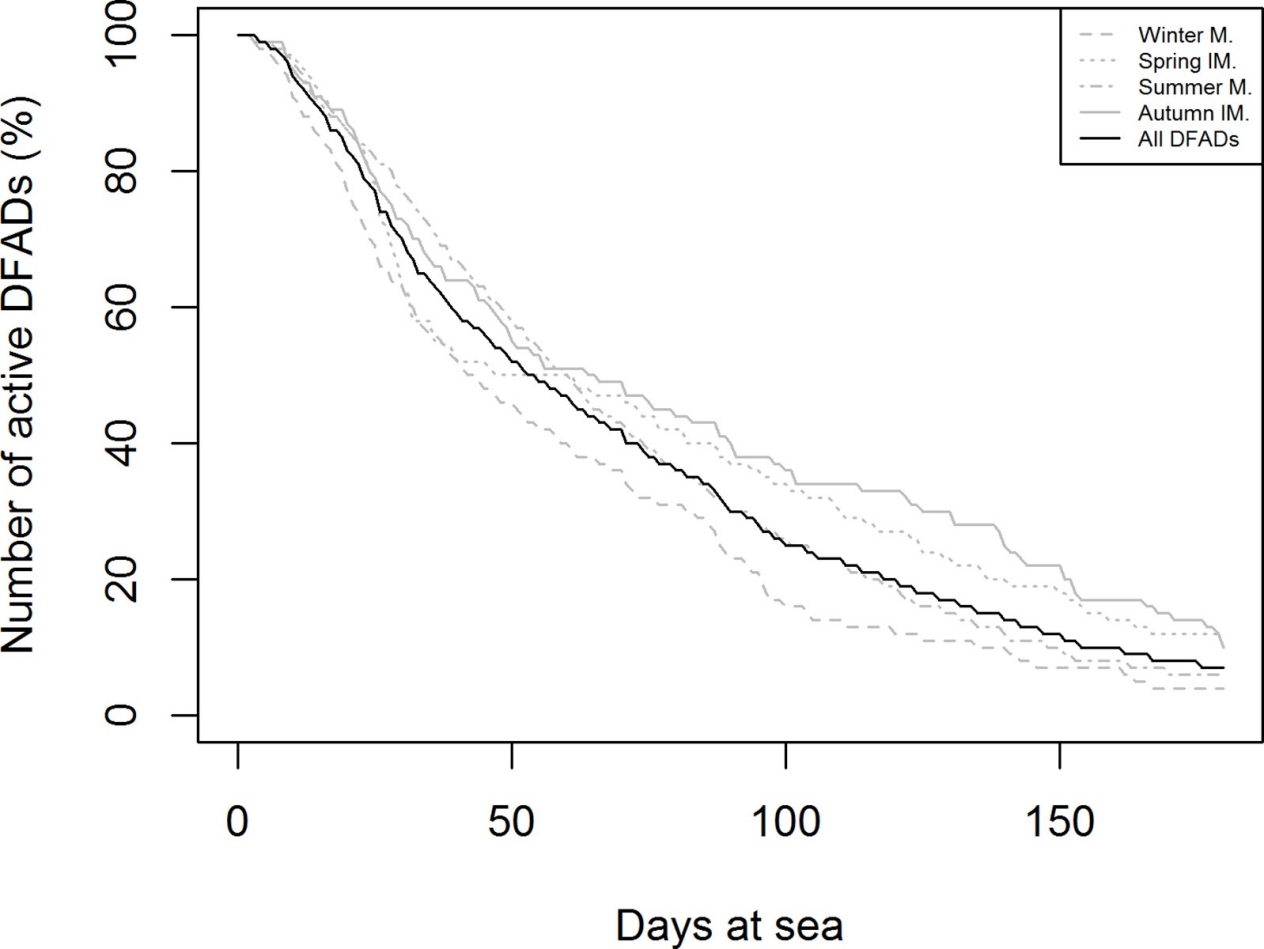
Percentage of DFADs available over 180 days for all DFADs (black line) and for DFADs deployed in different seasons (grey lines).

Although at the beginning a maximum limit of 180 days was established for the analysis based on the regular DFADs lifespan in the area [23] a preliminary data distribution analysis showed that after 60 days only ~50% of the objects were available for analysis **(Fig 1)**. As we want the modeled data to be representative of the general processes occurring at DFADs, the study was limited to the first 60 days of the DFAD trajectory.

### Data analysis

FAD logbooks contained information on the depth and material used to construct the underwater part of 776 DFADs. According to the FAD logbook, all the underwater parts of the DFADs were constructed with fishing nets and the depths ranged between 10 and 60 meters. These figures are in agreement with the regular depths used by the fleet in the Indian Ocean [37], although the most common average and depth for underwater parts is 15–20 m in the area [37]. Because it is believed that the DFAD structure may influence the detection capabilities and aggregation process of tuna and non-tuna species, DFADs were grouped in two categories: (i) shallow (i.e. < 20 meters) and (ii) deep DFADs (i.e. > 20 meters).

**Fig 2:**
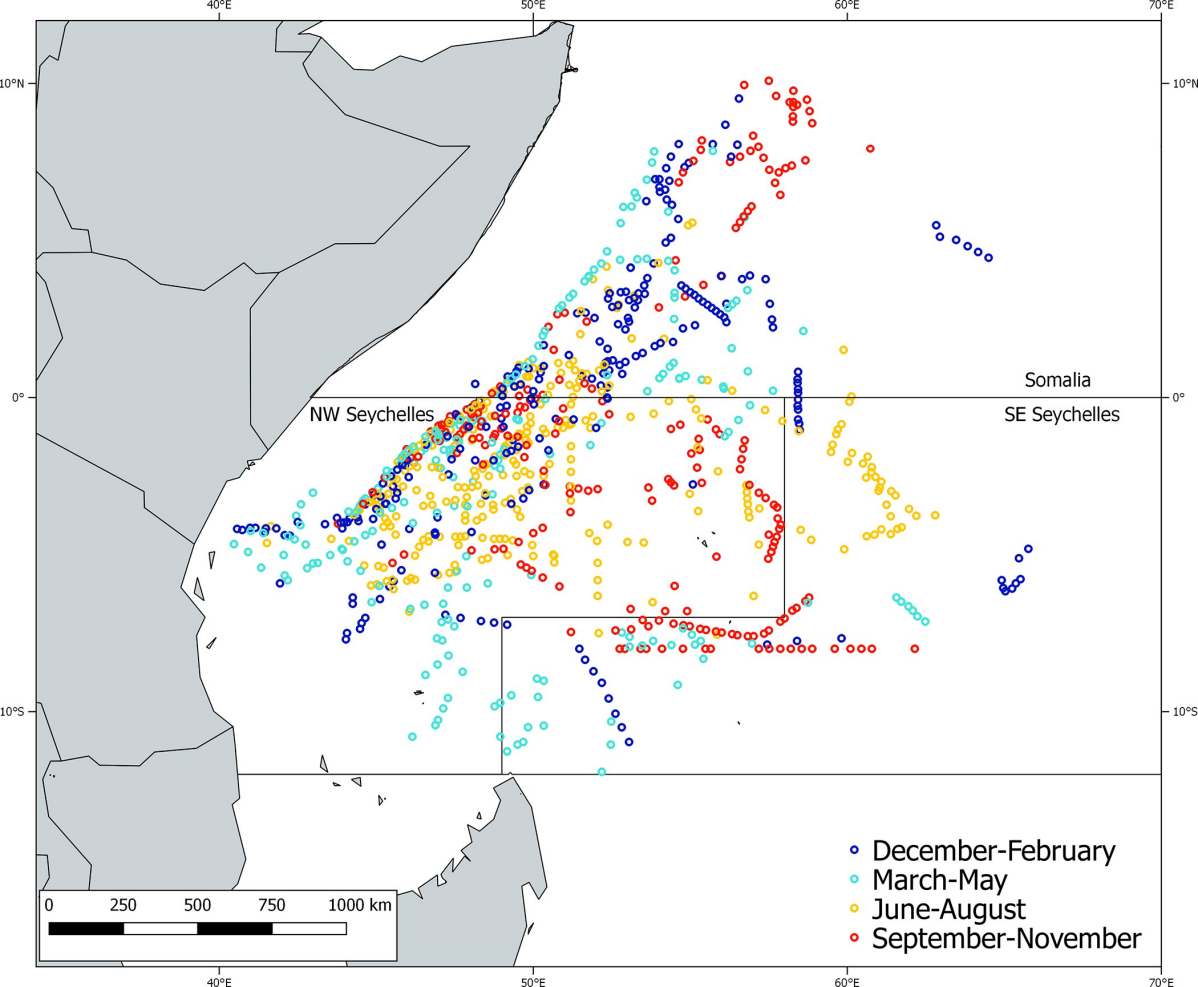
Spatial distribution of the 962 deployments of virgin DFADs for the different quarters.

Although the French fleet shows a strong seasonal pattern on deployment areas in the Indian Ocean [38], this was not observed in our data (**Fig 2**). Therefore, and due to the marked Indian Ocean monsoon system, the deployments were grouped according to the four different regimes that affect the oceanography and production in the region: (i) winter monsoon from December to March, (ii) spring intermonsoon from April and May, (iii) summer monsoon from June to September and (iv) autumn intermonsoon from October to November [39]. Moreover, to account for potential spatial differences in the aggregation process we applied the models by areas. The regions were based on the ZET (zones d'echantillonnage thonière) areas defined by Petit, Pallarés [40]: (i) Somalia, (ii) NW Seychelles and (iii) SE Seychelles. This spatio-temporal segregation of the data would help identifying possible differences in the aggregation process of the DFADs by area and season.

It is noteworthy the absence of deployments in Mozambique Channel. Although DFADs are used in this area, none of our identified deployments were in this area. This is due to several reasons (i.e. the higher proportion of natural objects in this area, inability to identify the buoy id, not being a first deployment of the object, etc).

The first day of detection, defined as the first day the buoy emits a non-zero signal for each species group, was also investigated to detect significant changes in the aggregation process. U Mann-Whitney tests were used to examine significant differences in the detection days for tuna and non-tuna species as well as by object depth category. Kruskal-Wallis H Tests followed by Dunn´s tests were used for multiple comparisons and to elucidate whether the first detection day differed between seasons.

Generalized additive mixed models (GAMMs) [41] with a Gaussian error distribution and identity link function, were established to analyze the trend of biomass over 60 days in virgin DFADs. The independent variable (i.e. days at sea) was included as the main parameter to construct the smooth term of the GAMM. The argument “by” within the splines was included to account for potential differences among periods, area and DFAD depth categories in the models. This implementation resulted in one independent smooth function being fitted for each monsoon period by area and for each DFAD depth category. Similarly, buoy identification code was included in the models as a random-effect term to address the dependency structure of the data (i.e. biomass abundance is collected repeatedly by the same buoy for each DFAD). In order to avoid model overfitting, maximum degree of freedom (k) was limited to k = 4 [42, 43]. Thus, the following notation was used to establish the final GAMM models:

Y∼s(daysatsea,k=4,by="area"/"depthcategory")+random=∼(1|ID_DFAD)

Where Y is the biomass of a fish group (i.e. tuna and non-tuna), s represents a penalized thin plate regression spline type smoother for days at sea, k is the maximum degrees of freedom allowed to the smoothing function and random = ~ (1 | ID_DFAD) is an ad hoc way of accounting for the autocorrelation structure of the data set in GAMMs.

GAMMs models were fitted using the gamm4 package [44] in RStudio [45]. The rest of statistical analysis were conducted using the package dunn.test [46].

## Results

### First detection day

**Fig 3:**
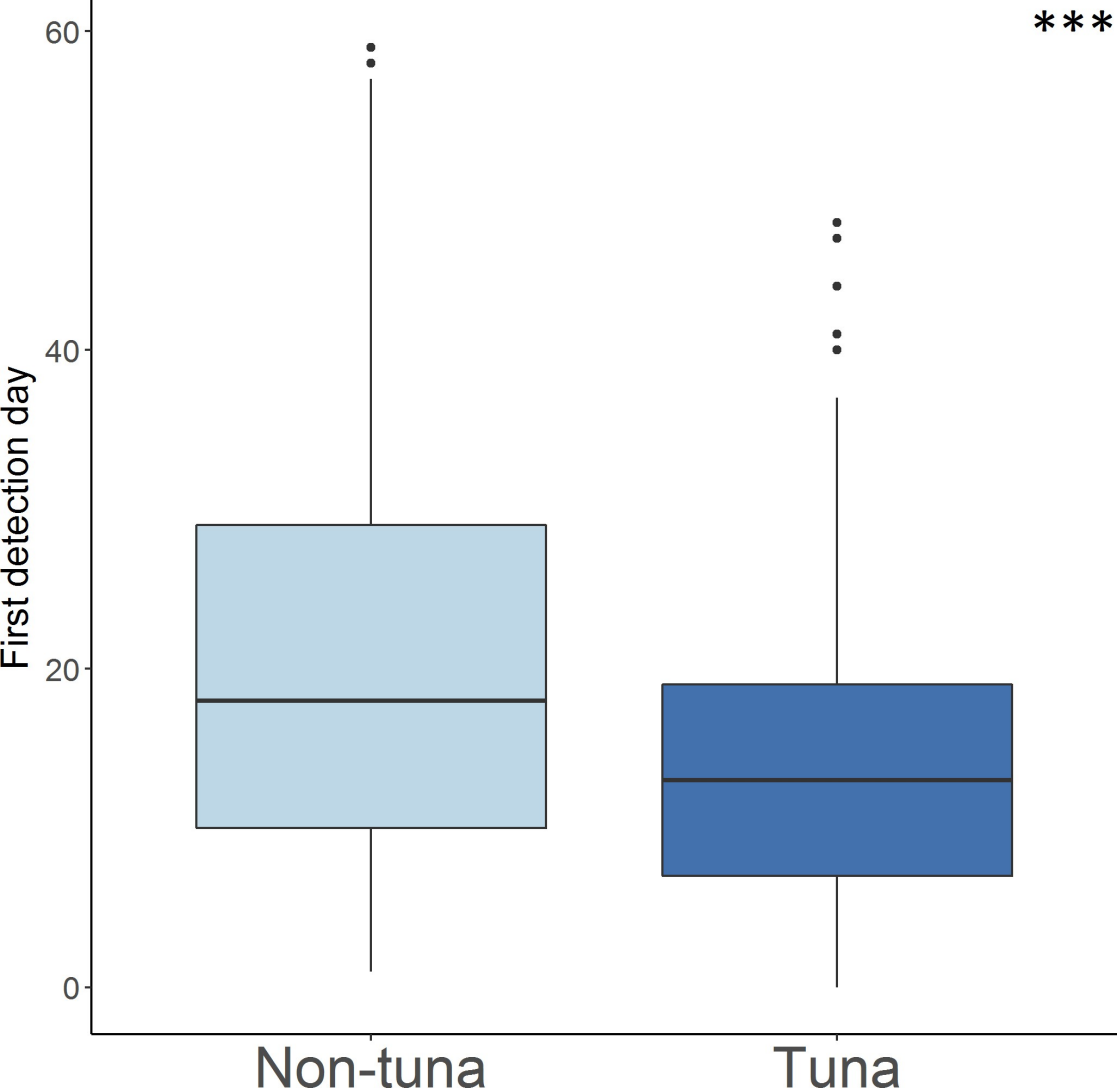
Boxplot of first detection day of tuna and non-tuna species to the object. Asterisks indicate the significance levels of differences following Mann-Whitney U test (* p < 0.05; ** p < 0.01; *** p < 0.001; NS not significant).

In general, the average period for the arrival of fishes to the DFADs (i.e. first day that the echo-sounder detected biomass) was 12.2±7.7 days. Tuna seemed to arrive at DFADs in 13.5±8.4 days whereas non-tuna species presence was recorded in 21.7±15.1 days, being differences significant between them (Mann-Whitney U test, *U* = 213980, *N*_1_ = 962, *N*_2_ = 962, *P* < 0.001) (**Fig 3, Table 1**).

**Table 1:** Mean and standard deviation of first detection day of tuna and non-tuna species according to DFAD depth and season (n = number of samples).

	n	MEAN+SD	
		TUNA	NON-TUNA
**General**	962	13.49±8.34	21.69±15.06
**Depth < 20m**	436	14.57±8.41	21.75±14.52
**Depth > 20m**	340	11.87±7.63	20.70±14.78
**Winter Monsoon**	304	12.26±8.08	19.92±14.50
**Spring Intermonsoon**	139	13.56±8.62	18.08±13.11
**Summer Monsoon**	366	14.01±8.37	23.13±14.86
**Autumn Intermonsoon**	138	14.77±8.40	25.18±16.70

**Fig 4:**
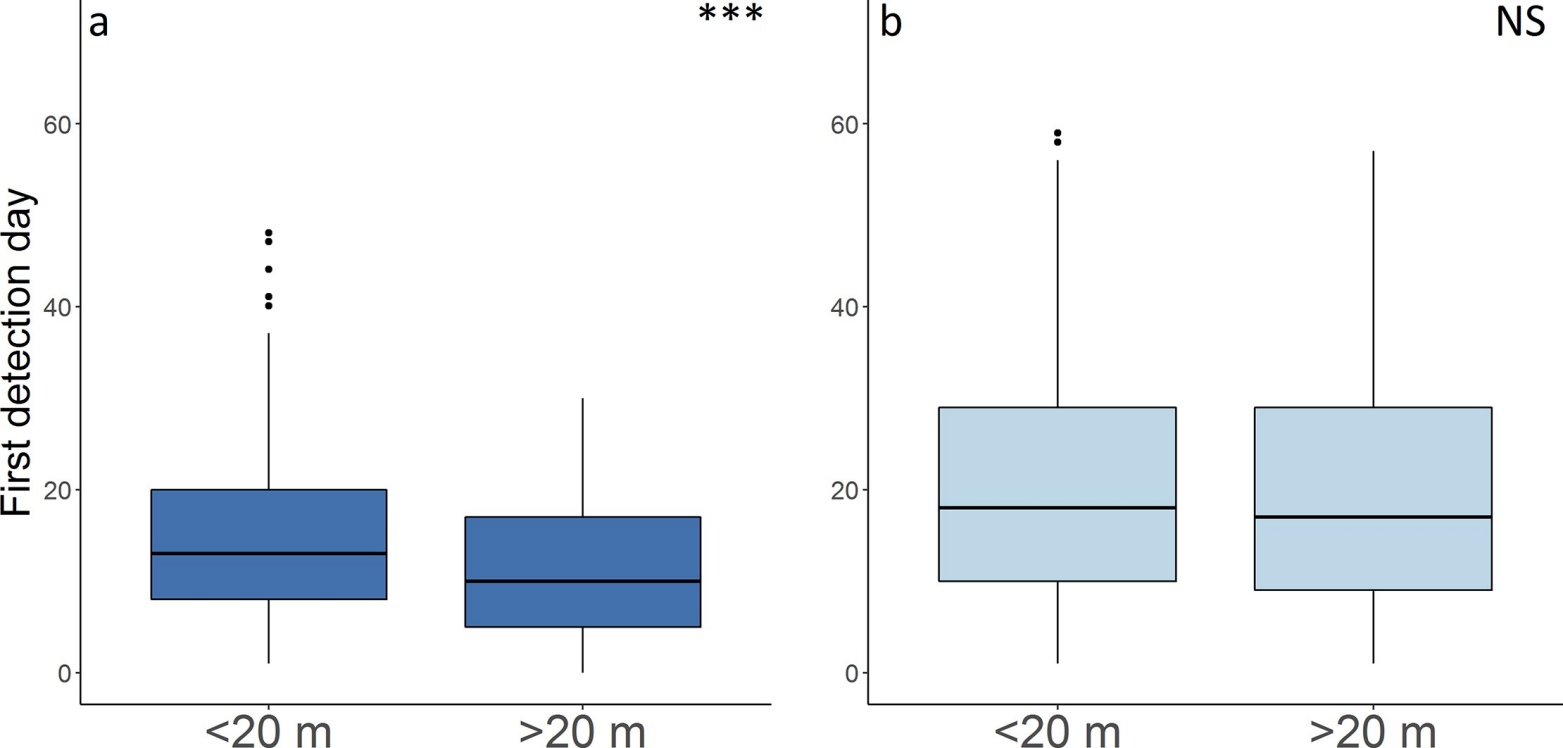
**Boxplot of first detection day to the object of (a) tuna and (b) non-tuna species for the different depth category of DFADs.** Asterisks indicate the significance levels of differences following Mann-Whitney U test (* p < 0.05; ** p < 0.01; *** p < 0.001; NS not significant).

The depth of the underwater part of the DFADs has species-specific effects. While for tuna species the depth of the underwater part of the DFADs was significant for an earlier detection (Mann-Whitney U test, *U* = 84334, *N*_1_ = 436, *N*_2_ = 340, *P* < 0.001), this difference was not significant for non-tuna species (Mann-Whitney U test, *U* = 36524, *N*_1_ = 436, *N*_2_ = 340, *P* = 0.318) (**Fig 4, Table 1**).

**Fig 5:**
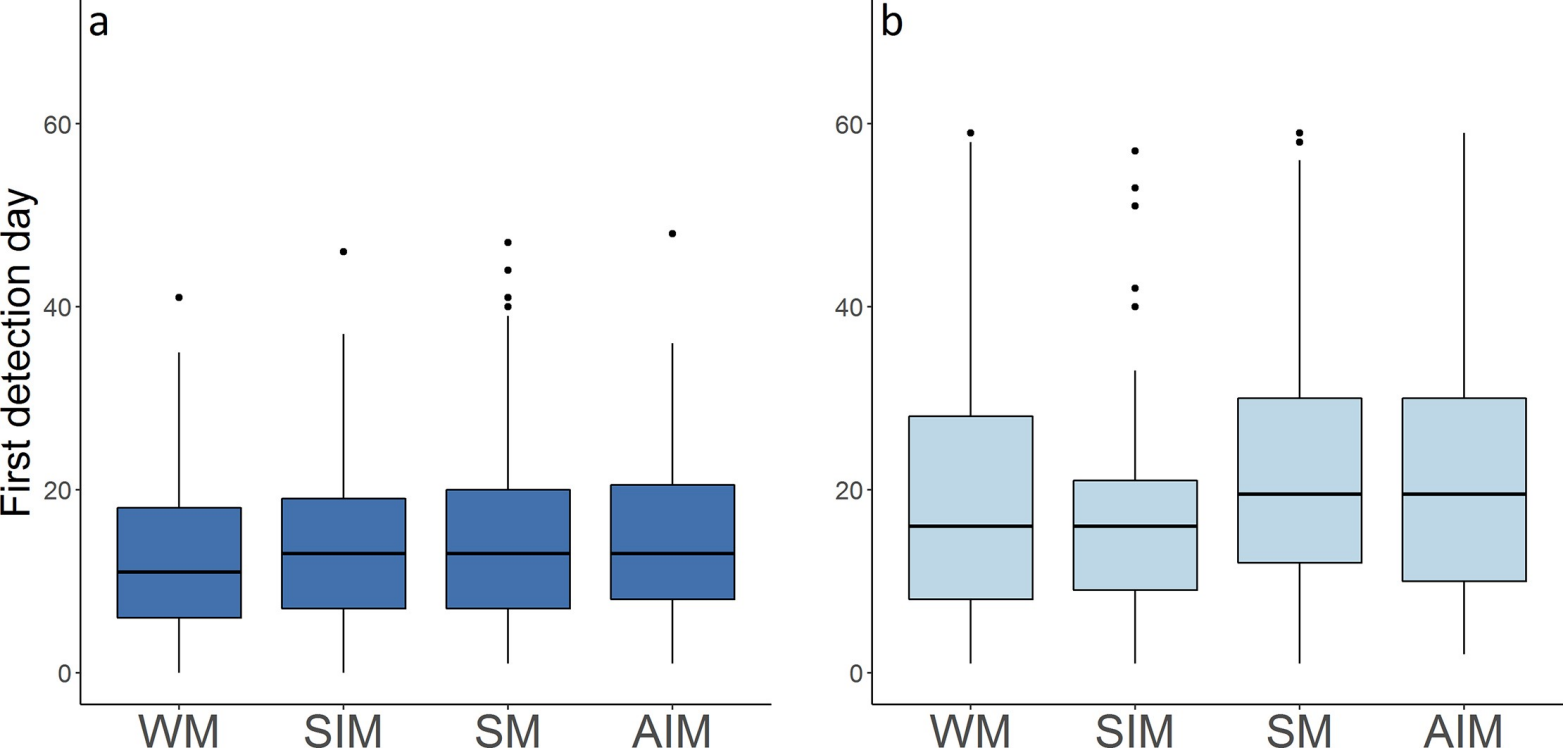
**Boxplot of first detection day to the object of (a) tuna and (b) non-tuna species by monsoon period.** WM = Winter monsoon, SIM = Spring intermonsoon, SM = Summer monsoon and AIM = Autumn intermonsoon.

The first detection day was also compared by monsoon period and species group (**Fig 5, Table 1**). Tuna was detected before non-tuna species in all cases. Significant season-specific differences were found for the first tuna detection day (Kruskal-Wallis test, *H*_4_ = 10.36, *P*<0.05). The Dunn´s Test confirmed a significant difference (*P* <0.05) between the winter monsoon and summer monsoon and spring/autumn intermonsoon periods. Non-tuna species also presented significant differences by periods (Kruskal-Wallis test, *H*_4_ = 15.45, *P*<0.05) and in this case, Dunn´s Test confirmed a significant difference (*P* <0.05) between winter monsoon and summer monsoon and autumn intermonsoon periods. Similarly, differences were found between spring intermonsoon period and summer monsoon and autumn intermonsoon periods.

### Aggregation dynamics

**Fig 6:**
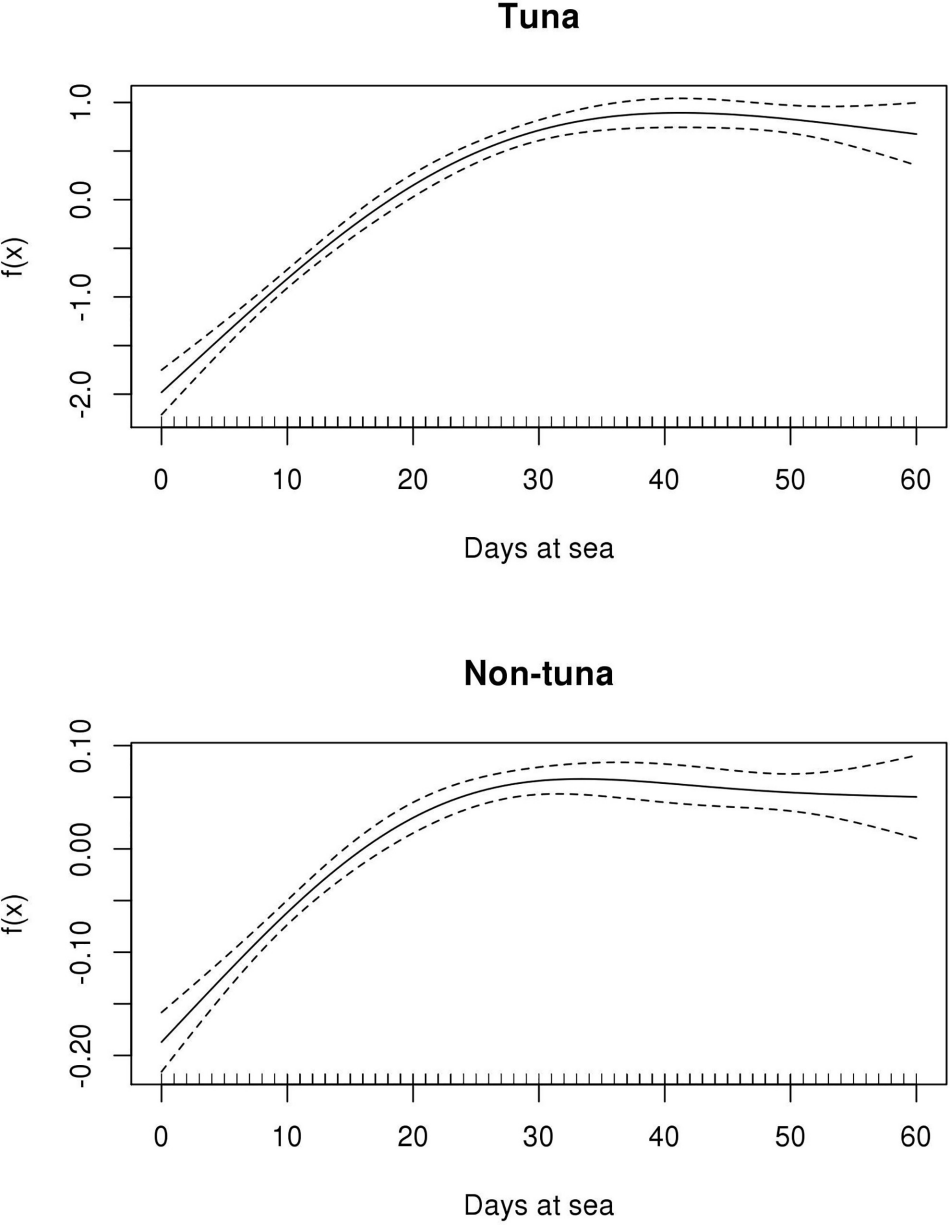
Functional shapes of the non-parametric relationship between biomass and days at sea with 95% confidence intervals (dashed lines), for tuna and non-tuna species.

The general models for the biomass aggregation of tuna and non-tuna species at DFADs appear to be similar (**Fig 6**). In both cases a clear increase in biomass was detected until approximately day 30. The biomass reaches a peak earlier in the case of non-tuna species, around day 30, while for tuna the peak is reached around day 40. After this period, both tuna and non-tuna biomass remained steady. **Table 2** presents a summary of the GAMMs and their parameter coefficients as well as the significance of the term days at sea for tuna and non- tuna species.

**Table 2:** Summary of GAMM models for tuna and non-tuna species.

	TUNA				NON-TUNA			
Parametric coefficients	Estimate	SE	z-value	p-value	Estimate	SE	z-value	p-value
Intercept	1.78	0.09	18.71	<0.001***	0.18	0.01	15.41	0.001***
**Approximate significance of smooth terms**	**edf**		**F-value**	**p-value**	**edf**		**F-value**	**p-value**
s(Days at sea)	2.87		152.6	<0.001***	2.85		75.02	0.001***

edf: effective degrees of freedom

*** Highly significant; s(), non-parametric smoother.

**Fig 7:**
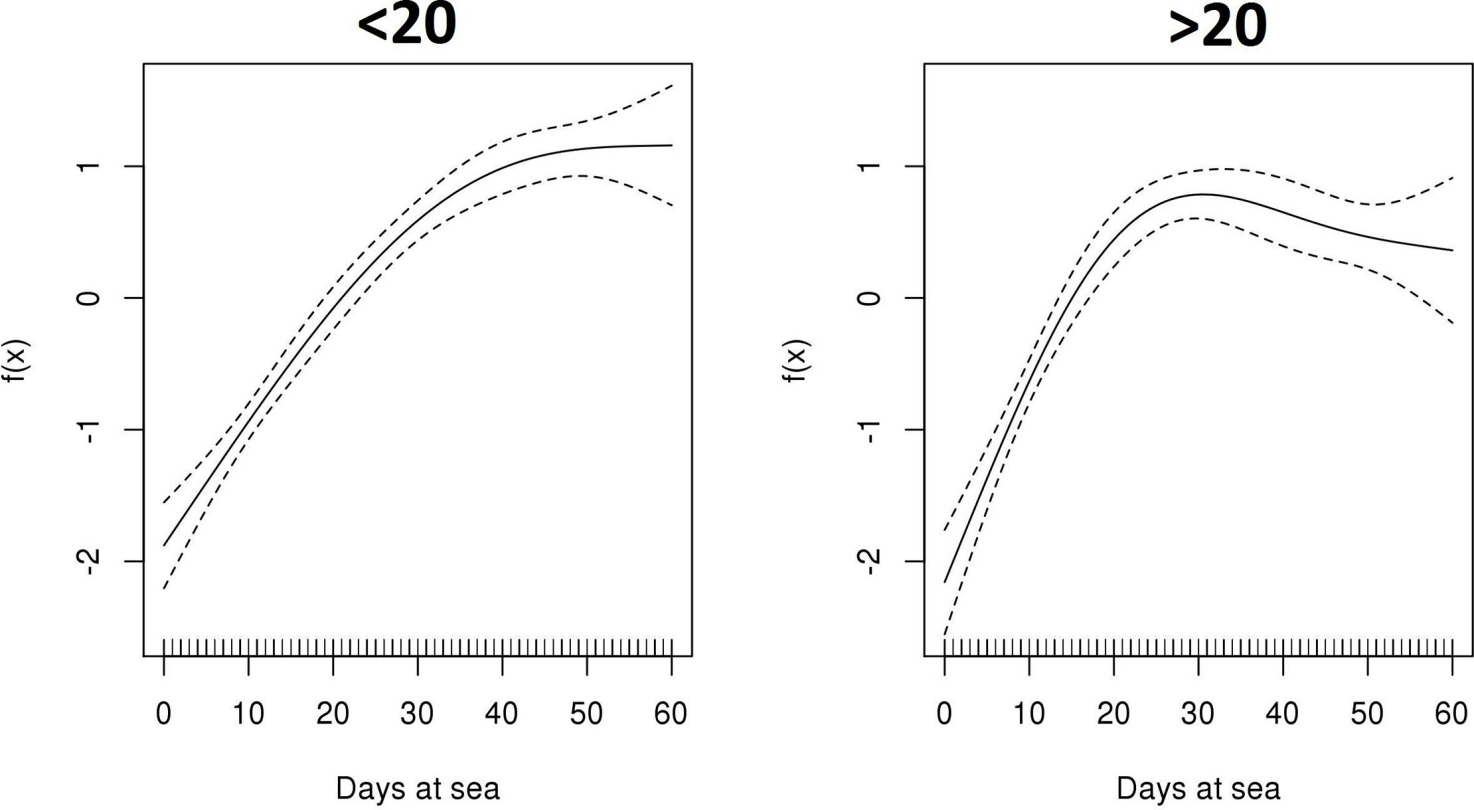
Functional shapes of the non-parametric relationship between tuna biomass and days at sea with 95% confidence intervals (dashed lines), according to the depth category of the object.

When modeling the tuna biomass according to the depth category of the object (**Fig 7**) the GAMM showed that deep objects reach the biomass peak almost 10 days earlier than shallow objects. Also, deep objects showed a biomass decrease after the peak while, in shallow objects, biomass remains stable after reaching the maximum. **Table 3** presents a summary of the GAMM and its parameter coefficients as well as the significance of the term days at sea by depth category for tuna species. For non-tuna species the aggregation dynamics on the first 60 days are very similar.

**Table 3:** Summary of GAMM models for tuna species according to the depth category of the object.

Parametric coefficients	Estimate	SE	z-value	p-value
Intercept	1.82	0.11	16.98	<0.001***
**Approximate significance of smooth terms**	**edf**		**F-value**	**p-value**
**s(Days at sea**_**depth <20m**_**)**	2.59		87.58	<0.001***
**s(Days at sea**_**depth >20m**_**)**	2.84		50.18	<0.001***

edf: effective degrees of freedom

*** Highly significant; s(), non-parametric smoother.

**Fig 8:**
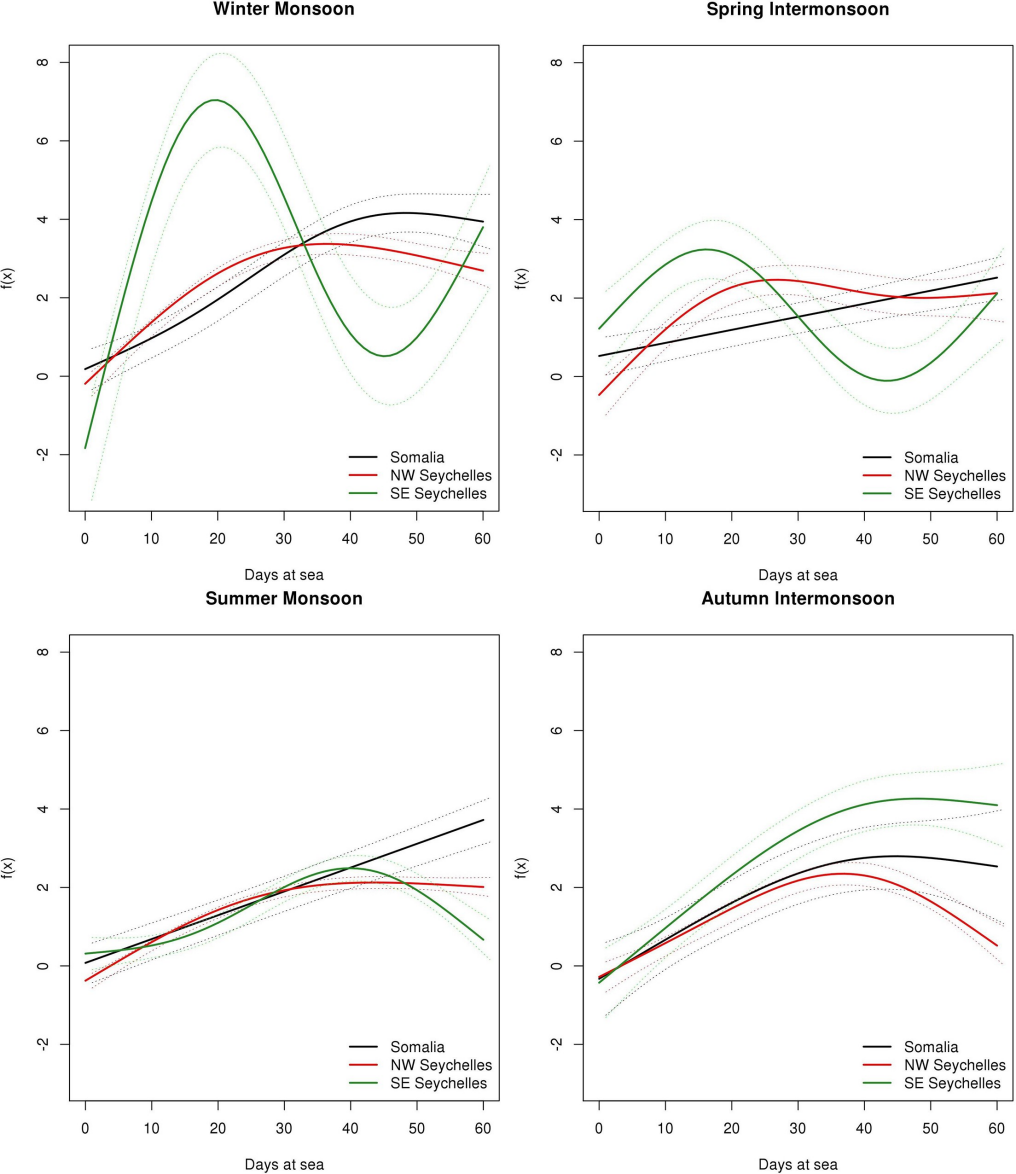
Functional shapes of the non-parametric relationship between tuna biomass and days at sea with 95% confidence intervals (dashed lines), for each period considered.

**Fig 8** shows a clear tuna biomass increase in all periods. Although there is not a great difference in the aggregation process by areas within a specific monsoon season, especially during the summer monsoon and autumn intermonsoon, the biomass aggregation process in the SE Seychelles area is slightly different during the winter monsoon and spring intermonsoon. In these seasons, a continuous increasing trend is observed during the first month followed by a strong decrease from day 30 onwards in SE Seychelles. In the same area, from day 40 onwards while there is a small decrease during the winter monsoon it stabilized during the autumn intermonsoon. In the case of Somalia and NW Seychelles, biomass trends are quite similar, with the exception of the spring intermonsoon, where NW Seychelles has a continuous increase while we find a biomass peak (i.e. day 25) in Somalia. In addition, the biomass trend shows a small decrease in the Somalia area after the first month in all periods. In the case of NW Seychelles, a stabilization around day 40 is shown from October to March, while from April to September it shows an increasing trend during the 60 days. **Table 4** presents a summary of the GAMM and its parameter coefficients as well as the significance of the term days at sea by season and area for tuna species.

**Table 4:** Summary of GAMM models for tuna species.

		Estimate+SE	edf	z-value	F-value	p-value
**Winter monsoon**	**Intercept**	2.38+0.22		10.8		<0.001***
	**s(Days at sea**_**Somalia**_**)**		2.66		38.73	<0.001***
	**s(Days at sea**_**NW Seychelles**_**)**		2.63		29.07	<0.001***
	**s(Days at sea**_**SE Seychelles**_**)**		2.96		30.70	<0.001***
**Spring intermonsoon**	**Intercept**	1.64+0.20		7.16		<0.001***
	**s(Days at sea**_**Somalia**_**)**		1.00		7.71	0.005**
	**s(Days at sea**_**NW Seychelles**_**)**		2.65		12.12	<0.001***
	**s(Days at sea**_**SE Seychelles**_**)**		2.78		4.35	0.009**
**Summer monsoon**	**Intercept**	1.44+0.12		11.19		<0.001***
	**s(Days at sea**_**Somalia**_**)**		1.00		77.60	<0.001***
	**s(Days at sea**_**NW Seychelles**_**)**		2.64		54.04	<0.001***
	**s(Days at sea**_**SE Seychelles**_**)**		2.61		5.58	<0.001***
**Autumn intermonsoon**	**Intercept**	1.74+0.23		7.86		<0.001***
	**s(Days at sea**_**Somalia**_**)**		1.78		3.34	0.02*
	**s(Days at sea**_**NW Seychelles**_**)**		2.46		6.85	<0.001***
	**s(Days at sea**_**SE Seychelles**_**)**		2.35		18.91	<0.001***

edf, effective degrees of freedom

*Significant

** Moderately significant

*** Highly significant; s(), non-parametric smoother.

**Fig 9:**
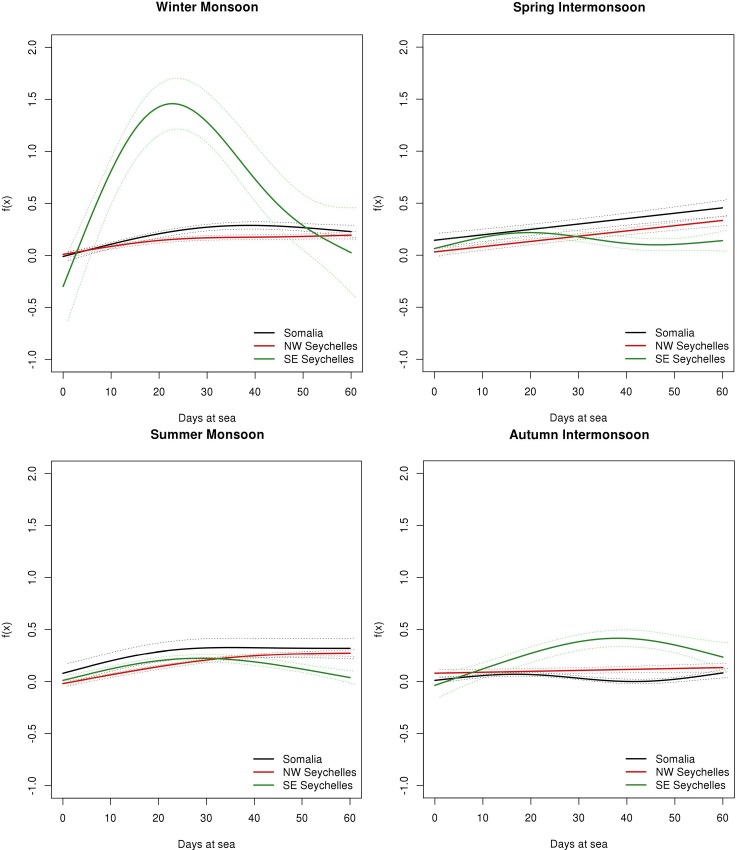
Functional shapes of the non-parametric relationship between non-tuna biomass and days at sea with 95% confidence intervals (dashed lines), for each period considered.

For non-tuna species (**Fig 9**), models also shown an increasing biomass trend over the 60 days but much smoother than in the case of tuna. In this case, SE Seychelles also shows the most different biomass aggregation trend, with a biomass peak at 25 days during the winter monsoon. Somalia and NW Seychelles models show a constant linear increasing trend. Table 5 presents a summary of the GAMM and its parameter coefficients as well as the significance of the term days at sea by seasons and area for non-tuna species.

**Table 5:** Summary of GAMM models for non-tuna species.

		Estimate+SE	edf	z-value	F-value	p-value
**Winter monsoon**	**Intercept**	0.21+0.03		8.54		<0.001***
	**s(Days at sea**_**Somalia**_**)**		2.26		11.54	<0.001***
	**s(Days at sea**_**NW Seychelles**_**)**		1.70		4.18	0.01*
	**s(Days at sea**_**SE Seychelles**_**)**		2.97		69.96	<0.001***
**Spring intermonsoon**	**Intercept**	0.19+0.02		8.36		<0.001***
	**s(Days at sea**_**Somalia**_**)**		2.51		6.69	<0.001***
	**s(Days at sea**_**NW Seychelles**_**)**		1.00		20.73	<0.001***
	**s(Days at sea**_**SE Seychelles**_**)**		2.13		0.87	0.56
**Summer monsoon**	**Intercept**	0.19+0.02		8.45		<0.001***
	**s(Days at sea**_**Somalia**_**)**		2.41		12.09	<0.001***
	**s(Days at sea**_**NW Seychelles**_**)**		2.43		59.95	<0.001***
	**s(Days at sea**_**SE Seychelles**_**)**		2.36		5.09	0.005**
**Autumn intermonsoon**	**Intercept**	0.14+0.02		5.62		<0.001***
	**s(Days at sea**_**Somalia**_**)**		1.00		0.05	0.83
	**s(Days at sea**_**NW Seychelles**_**)**		1.00		0.55	0.45
	**s(Days at sea**_**SE Seychelles**_**)**		2.54		10.51	<0.001***

edf, effective degrees of freedom

*Significant

** Moderately significant

*** Highly significant; s(), non-parametric smoother.

## Discussion

Many studies have investigated the behavior of tuna [22, 36, 47–51] and non-tuna species [29, 32] at different spatio-temporal scales around FADs but few of them have investigated the aggregation process of species in detail [4, 24, 34, 52]. This is the first-time, to our knowledge, that fishery independent data provided by fishers´ echo-sounder buoys have been used to explore at a fine- and meso-scales the aggregation processes of species at virgin DFADs in the Indian Ocean. The effect of the DFAD depth was assessed in relation to the first detection day and the aggregation dynamics, finding significant differences for tuna and not-significant effects for non-tuna species. The aggregation dynamics of tuna and non-tuna species generally showed an increasing trend during the first 30 days after deployment, but also showed specific seasonal and area differences per group. These differences suggest that tuna and non-tuna species may have different associative behaviors.

In recent years the potential use of DFADs as scientific platforms has been highlighted by the scientific community [26, 27]. Indeed, echo-sounder buoys represent a very powerful tool to continuously and automatically collect and stream information on thousands of DFADs in a very cost-effective manner. Considering that around 100,000 objects may be deployed annually worldwide [37, 53, 54], these devices can provide acoustic data on the biomass of tuna and non-tuna species underneath them from all tropical oceans on a regular and effective basis. Fishers echo-sounder buoys have been very useful for the investigation of the aggregation process of tuna and non-tuna species at DFADs, so these data could also allow scientists to better assess several issues of scientific relevance related to DFADs, for example they could investigate the spatio-temporal distribution in relation to environmental parameters or could produce a fishery independent abundance index for stock assessment [55], among others.

The echo-sounder buoys used in this study provide a single biomass value without determining the species or size composition of the fish underneath the DFAD. Tuna are well known to engage in both horizontal and vertical movements around DFADs [51, 56–58] and, although overlap may exist in the vertical range of the three species of tuna, tagging data suggest differences in depth preferences between the species and the different sizes of these species. Skipjack tuna schools tend to remain in shallower waters, as do small yellowfin and bigeye tuna that are found occupying similar depth ranges as skipjack. However large individuals of bigeye and yellowfin tuna are found at greater depths. This potential segregation has also been observed in the Indian Ocean by dedicated surveys around DFADs using scientific echo-sounders [33]. In addition to this segregation by species and sizes, the vertical distribution of tuna at DFADs may vary depending on different factors, including oceanographic conditions (e.g. thermocline depth or surface and subsurface currents) [51, 56], total associated fish biomass or number and size of species present at DFADs [36]. Recent acoustic research [59] has found a different frequency response for skipjack compared to bigeye and yellowfin tuna when analyzed simultaneously using multiple acoustic frequencies, based on anatomical differences (i.e. the skipjack does not have a swim bladder while bigeye and yellowfin tuna do). Incorporating multi-frequency technology in echo-sounder buoys could help in more effective tuna species discrimination at DFADs, with obvious advantages for science and management. Having specific composition information before the fishing set could help to mitigate, for example, the catch of small bigeye, which is one of the most important concerns associated with DFAD fisheries in tunaRFMOs [18], thereby contributing to a more sustainable exploitation of the resources. Future studies should consider improved methodologies and buoy technologies to effectively discriminate between the three species of tropical tuna so that species-specific aggregation processes can be better understood.

In addition to this spatial competence by tuna species and sizes, overlap may also exist between tuna and non-tuna species that occupy different depth layers depending on the time of day or the area. To take this consideration into account, several options were considered during preliminary analyses to choose the time at which the acoustic signal is more representative of the biomass around the DFAD. For example, model the diel biomass estimated by the echo-sounder using general additive models and choose the maximum biomass value between the peak hours. Another option was to choose the sample with maximum biomass value around sunrise (between 3 a.m. and 8 a.m.), since this is the time when tuna and other species are supposed to be more closely aggregated under the DFADs [60–63]. However, due to sampling constraints (i.e. sampling frequency is not hourly), using these options would considerably limit the number of samples available in this study. Future studies with more data should consider the use of the above-mentioned options and compare results with current findings. Also, the position of the fish in relation to the echo-sounder beam and its detectability is something to be considered. Some bycatch species are known to be more strongly and tightly attached to the DFAD (intranatant/extranatant species, see [64]) than tuna species (circumnatant) and thus, may be more easily detected. Besides, some species like tuna make longer excursions out of the DFAD when compared to most non-tuna species [24, 31, 34]. However, the cone shape of the beam of the echo-sounder, with a larger area with increasing depth, compensates the detectability of tuna individuals. Ideally, future studies should combine echo-sounder buoy sampling around DFADs with dedicated tagged and monitored fauna to infer detectability rates per species and size and assist in acoustic signal interpretation.

### First detection day

Our research shows that, in general, the average first detection of fish at DFADs occurs at around 1–2 weeks (~12.2±7.7 days). Previous studies in the field have shown different results. Some works observed a faster colonization of DFADs by tuna, generally in less than a week [52] while others have suggested similar aggregation periods. For example, fishers working with anchored FADs (AFADs) in the Philippines wait 11 days after the first deployment before checking for biomass aggregation [49]. AFADs may be easier to detect and colonize as they are fixed reference points located in areas close to the coasts. In general, the first group to be detected by the echo-sounder appear to be the tuna. Some authors, based on interviews with fishers and other investigations, have stated that non-tuna species may arrive first at DFADs [1, 4, 23]. For example, fishing masters of the purse-seine fleets working in the Western Indian Ocean suggested that 1–3 weeks are necessary to colonize virgin DFADs by non-tuna species [23]. However, for tuna, half of them said that aggregation process may not be time-dependent, and the other half said that at least one month is needed. Our results, using fishery-independent data from continuously monitoring DFADs since their first deployment, showed that this time may be shorter than expected. A possible explanation for this is that at the time of the interviews (i.e. 2007) they had just started working extensively with DFADs equipped with echo-sounders and, hence, were not able to accurately interpret echo-sounder buoy information in general, and in particular related to the aggregation process. Another possible explanation is that non-tuna species may be easier to observe than tuna when fishers approach the object, as non-tuna species are strongly associated with DFADs [65] showing less frequent excursions out of DFAD [23–25, 34]. Future studies should consider consulting fishers again on this issue in order to determine whether their beliefs have changed through time due to the extensive use of these technological devices or fishing strategy changes. Indeed, fishing strategy changes may have been occurring in recent years in the Indian Ocean (i.e. smaller soak times before first setting) to try to reduce the potential steal rate of DFADs, and consequently, catches.

Although our results for the first detection day slightly differ from some of the previous works, the differences could be partially explained by the minimum detection threshold of around one ton that buoys have. If the amount of fish aggregated under the DFADs is lower than one ton (as for the conversion done by the manufacturer) during the first few days the object is at sea, the echo-sounder may not be indicating biomass presence, potentially biasing the analysis. This limitation may be significant in the case of non-tuna species, since bycatch species are normally found in lower amounts than tuna species at DFADs (i.e. in a range between 1–5 tons [25, 66, 67]). However, we do not expect this to have implications for the final results as tuna usually swim in large schools [68].Moreover, Satlink buoys use a method that converts raw acoustic backscatter into biomass using an empiric algorithm based on the target strength and weight of skipjack tuna, which is the main target species of the DFADs’ purse seine fishery. Whereas the skipjack tuna does not have a swim bladder, the majority of bycatch species do. Swim bladder species normally produce a much higher echo than bladderless ones since this hydrostatic organ, when present, is responsible for 90–95% of the backscattering energy [69]. Accordingly, one ton of the skipjack-based algorithm biomass may not necessarily represent the same amount of non-target species, but probably less, and thus the one ton threshold may not weaken the results and conclusions of this work. Still, it would be very useful to be able to work without this detection threshold. To overcome this, there is a need to propose collaborations between buoy companies, fishers and scientists to develop a process for a routine, continuous, confidential and RFMO-supported raw data transfer, with reasonable delays, between the purse seine industry and research centers. Access to these raw data would provide scientists information without the threshold limitation, which could help in the investigation of sustainability and the management of the exploited resources.

### Influence of DFAD structure on the aggregation process

DFADs’ characteristics can vary between fleets and oceans but in general they are composed of a raft and an underwater part hanging below the object [2, 70]. The depth reached by the structure ranges from 15 to 80–100 meters and is ocean-specific (15-20m in the Indian Ocean, 80-100m in the Atlantic Ocean and around 30m in the Eastern Pacific Ocean) [37], although in recent years a trend toward deeper objects in all the oceans has increased [71]. The study has suggested a significant relationship between object depth and faster tuna aggregation. For non-tuna species, on the contrary, this relationship appeared not to be significant. These differences may be related to the vertical distribution of the species under DFADs. As non-tuna species usually occupy shallower waters (<25 meters) [34] the depth of the underwater part may not affect their detection ability, and consequently, the aggregation process. It may happen, however, that the shape or material of the floating part would be more important than the hanging structure in the aggregation of non-tuna species. Similar works should study non-tuna species aggregation process differences on DFADs with different features of the floating structure. By contrast, tuna are usually found in deeper waters (i.e. > 25m) [34, 50, 51, 72, 73], and thus, deeper objects might be easier to detect visually. Indeed, the eye is considered the main sense organ of tuna which is better adapted to low light [74, 75]. However, other senses may also help in detecting the object (e.g. sound, chemical) [76, 77]. Although the results of this study may suggest that the depth of the underwater structure could play an important role in the aggregation of tuna at DFADs, further studies should investigate in detail the aggregation-segregation phenomenon in relation to sensory cues, as understanding the key drivers of the associative behavior is crucial for the adequate management of exploited resources.

During the first 30 days of the object at sea, the tuna biomass follows the same biomass increasing trend for both DFAD depth categories. However, from that point onwards, there is a decrease of the tuna biomass in deep DFADs. The reasons causing the loss of tuna biomass in deeper objects after the first month are difficult to determine, as there is little evidence and information on the factors driving fish to leave the DFAD. Several hypotheses have been generated by the scientific community to explain why tuna and non-tuna species associate with DFADs [1, 6, 7] but none to elucidate why fish leave them. The interviews conducted by Moreno et al. (2007) with fishers in the Indian Ocean, pointed out a change in the speed or direction of the drifting object, the presence of large predators, or DFADs entering the continental shelf as the main reasons for fish to leave a DFAD. Future research should combine echo-sounder buoy data along the trajectory of the object with environmental information to investigate the drivers of biomass dynamics around DFADs.

To our knowledge, this is the first time that the depth of the net hanging down has been related to the aggregation process. So far, the effect of the depth has been investigated in relation to the tuna catch species composition. Lennert-Cody and Hall [78] and Lennert-Cody, Roberts [79] found that the species composition of the catch varied with the depth of the net hanging down, suggesting there could be more successful bigeye and yellowfin sets on objects with deeper structures in the Eastern Pacific Ocean. However, similar works in the western Pacific Ocean [80] did not find such a positive relationship for bigeye tuna. Related to these investigations, the Inter-American Tropical Tuna Commission considered the possibility of limiting the depth of the underwater part hanging below the FAD [81] in order to reduce the bigeye bycatch, although the measure was not ultimately adopted and it is not being implemented by the fleet. Therefore, since in this study we have found evidence that the depth of the object’s underwater part affects the aggregation of tuna, further studies should be proposed to help to design specific management measures for tuna species.

### Aggregation process by periods and areas

The large quantity of data available for this work (42,322 daily biomass observations) provides a high spatio-temporal resolution to study the aggregation process across different seasons. The Indian Ocean is characterized by strong environmental fluctuations associated with monsoon regimes that affect ocean circulation and biological production [39]. Changes in biophysical factors associated with seasonality (i.e. chlorophyll, temperature, salinity, dissolved oxygen) may play an important role in the aggregation dynamic of tuna and non-tuna species. Our first detections were consistent for all the monsoon periods analyzed (i.e. tuna were always detected earlier than non-tuna species at DFADs). The first detection days of tuna species at DFADs were statistically different across seasons, with a maximum difference of 2.5 days. Despite this result, when the analysis was made between seasons, we only found differences for arrival time among the winter monsoon and summer monsoon and autumn intermonsoon periods. In relation to the aggregation dynamics, the maximum biomass is reached later (i.e. after 40 days at sea) in summer and autumn periods in comparison to other seasons. These differences may be related to changes in productivity between periods. A strong upwelling occurs in the Western Indian Ocean in the summer monsoon season [82], where cold and highly saline waters with a high nutrient content are pumped up, producing an increase of primary production along the coast of Somalia [83, 84] which can spread more than 500 km offshore [85]. This productivity increase may make floating objects less attractive for tuna as enough prey concentration may be available in the surface environment. On the other hand, productivity is significantly lower during the winter monsoon [86], where not only both tuna and non-tuna species arrive earlier at the DFADs in comparison to other seasons, but the biomass also peaks earlier compared to the summer and autumn periods. It is known that the distribution of large predators, like tuna, is affected by productivity [87], but the relationship between productivity and DFAD attractiveness remains unclear. This work hypothesizes that productivity and DFAD attractiveness may be inversely related. Future research at different spatio-temporal scales should link acoustic information with remote sensing variables, including chlorophyll-a, to analyze whether, and how, environmental conditions are related to the aggregation dynamics of tuna and non-tuna species at DFADs.

It is important to highlight the different trends that have been observed in the SE Seychelles from December to April-May, while for the rest of the year all areas showed similar trends. During the winter monsoon the fishery is usually located in the SE Seychelles and Chagos areas in search of FSC [88], where schools of yellowfin and bigeye tuna happen to be feeding or spawning in surface waters [89]. The difference in the observed aggregation dynamics in the SE Seychelles area coincides with the FSC fishery season, which could affect the species composition in the area and, hence, the aggregation dynamics around DFADs.

In addition to environmental features, other factors could also affect the aggregation process of tuna and non-tuna species at DFADs, such as the density and abundance of the population and of the DFADs. Robert, Dagorn [36] suggested that social interactions underlie aggregation processes. Because tuna biomass seems to increase after the deployment of a DFAD, we could interpret this to mean that tuna associative behavior may be dependent on fish density [90], and thus, an increasing abundance of fish at DFADs could lead to stronger attraction and retention behaviors [24]. Moreover, an overall saturation point observed after approximately 30–40 days may indicate that after a certain specific period, the aggregation of tuna could be compromised. Therefore, DFADs could contribute to the formation of large schools and the optimal school size [91] during the first 30–60 days at sea.

## Conclusions

Fishing around DFADs has become the main strategy used by tropical tuna purse seiners in recent years [11]. The concern surrounding DFAD fishing generally comes from the uncertainty about the impacts it produces (e.g. a higher level of bycatch, alteration of tuna movements, impacts on the habitat, etc.). To consider new and alternative management options, it is necessary to improve our knowledge of the key factors driving the species aggregation-segregation processes with floating objects. This study contributes to better understanding of the tuna and non-tuna aggregation mechanisms in relation to both the DFAD structure and deployment seasons. In summary, the first detection day of fish at DFADs was around 1–2 weeks and differed significantly between tuna and non-tuna species. Although fishers consider that deeper DFAD may favor faster and larger fish aggregations [2], this aspect has never been investigated in detail in relation to the aggregation processes. The analysis showed a significant relationship between object depth and colonization of tuna, suggesting a faster tuna colonization for deeper objects. For non-tuna species this relationship appeared not to be significant. The Indian Ocean is characterized by strong environmental fluctuations associated with monsoon regimes and seasonal variability in fishing grounds [92] and catch [93]. Therefore, analyzing the aggregation process in different periods could help with designing spatio-temporal management measures for tuna fisheries. The aggregation dynamics differed between monsoon periods in both tuna and non-tuna species. These differences could be explained by changes in the biophysical environment associated with seasonality, although there may be other social factors affecting the aggregation process of tuna and non-tuna species at DFADs, such as the density and abundance of the local tuna population or DFADs. The results of this research can be used to assist in working for the sustainability of tuna fisheries and may help to design management measures for tuna and non-tuna species, such as optimization of fishing activities.
